# Case of Peri-Uterine Cellulitis

**Published:** 1885-12

**Authors:** Chas. L. Gwyn

**Affiliations:** Galveston, Texas


### CASE OF PERI-UTERINE CELLULITIS WITH SEQUELÆ.

*Reported to Galveston County Medical Club September 7th*, 1885,
*By Chas. L. Gwyn, M. D., Galveston, Texas.*

For Daniel's Texas Medical Journal.

CASE :—April 2nd, 1885, Ella H., age 36 years, white, married, sterile.

The above patient has hitherto been a patient for constitutional syphilis of sixteen years standing; has lost the left *ala* of her nose and is very stout for her heighth.

A speculum examination some months ago revealed an enlarged uterus, but whether from hyperplasia or from some enclosed tumors I could not determine, as I had no uterine sound at hand; the size was that of a moderate sized fœtal head. The cervix and os externus were normal; she complained of more or less dysmenorrhœal pain preceding and during her menses.

For the two or three months preceding this attack, her general health had so much improved that, from motives of economy, she undertook the washing for her own family. The to and fro motion of her body (the abdomen being very large) threw the already enlarged uterus, with each motion of the body, against the brim of the pelvis. Instead of desisting from her work she presisted in the it until it was done. I was not called in until the next day, when I found her suffering from intense abdominal pain; knees drawn up; heat, swelling, and pain in the vagina and at the vulva; could not bear pressure over the uterus; color of complexion icterous; temperature 104.6.

For several days the symptoms varied but little, and I had to have recourse to the hypodermic syringe to allay the intensity of the pain.

Suppuration was ushered in by a severe rigor; the abscess pointed, and was discharged into the rectum anteriorly, and just above the internal sphincter. Apparent convalescence took place, and in a few days the patient, without permission, left her bed, and attempted to resume her household duties. Her menses appeared, and from the intense pain she had to take her bed during the next thirty-six hours. She passed four mucoid polypi about two inches long, and about three-quarters of an inch in diameter, irregular in shape from compression. Decomposition had commenced in them. They were followed by clots of blood of varying size, and a feotid discharge resembling the lochia in character and odor. The uterus could no longer be felt above the pubes, and digital examination demonstrated that it had reduced to two-thirds, or one-half of its former size.

A day or two elapsed, and pain commenced in the right iliac region; a distinct tumor commenced to form, and for a day I was undetermined whether it was from obstruction of the colon or an ovaritis; but cathartics made their way through, while the tumor enlarged. Towards the close of the case, partial obstruction did take place from pressure. There was a tendency for it to point first alongside of the vulva, then between the vulva and anus, and it did eventually break into the rectum about the same place that the metric abscess broke. The stomach was intensely irritable during the whole attack, rejecting foods, medicines and fluids. I was considerably alarmed after a hypodermic injection, she being very fleshy, I was not able to see the course of the veins, and accidentlly gave an intravenous injection of about half drachm of fluid, containing half grain of sulphate morphine; pain immediately shot up the arm, the neck, and down to the heart, producing a spasm of that organ, simulating angina pectoris, then a gastralgia, accompanied by vomiting and retching. Strong whisky relieved her temporarily, but the spasms recurred at intervals during the day. Since the above accident, each hypodermic injection has been followed by slight cardiac spasms regardless of the location of the puncture. Nussbaum records an entirely different set of symptoms for intravenous injections of morphia.

By use of tonics, etc., the patient made a rapid recovery and is in better health than for years before.

---

A NEW ETIOLOGICAL FACTOR.—Our Doctor Merrriman denies that strabismus is due to a "*micro-cock-eye.*" He says that a number of recent cases, at least, have been produced by frantic efforts to "see the point" of some of *The Age's* jokes.

